# Epidemiological and Molecular Investigation of Feline Panleukopenia Virus Infection in China

**DOI:** 10.3390/v16121967

**Published:** 2024-12-23

**Authors:** Yinghui Wen, Zhengxu Tang, Kunli Wang, Zhengyang Geng, Simin Yang, Junqing Guo, Yongzhen Chen, Jiankun Wang, Zhiyu Fan, Pengju Chen, Jing Qian

**Affiliations:** 1 College of veterinary medicine, Henan University of Animal Husbandry and Economy, Zhengzhou 450046, China; 2 Key Laboratory of Veterinary Biological Engineering and Technology, Institute of Veterinary Medicine, Jiangsu Academy of Agricultural Sciences, Nanjing 210014, China; 3 Henan Institute of Modern Chinese Veterinary Medicine, Zhengzhou 450002, China; 4 Nanjing Taihe Bioengineering Co., Ltd., Nanjing 210014, China

**Keywords:** feline panleukopenia virus, VP2 gene, genetic evolution, molecular characterization, 91S substitution

## Abstract

The feline panleukopenia virus (FPV) is a highly contagious virus that affects cats worldwide, characterized by leukopenia, high temperature and diarrhea. Recently, the continuous prevalence and variation of FPV have attracted widespread concern. The aim of this study was to investigate the isolation, genetic evolution, molecular characterization and epidemiological analysis of FPV strains among cats and dogs in China from 2019 to 2024. The 41 FPV strains, including 38 feline strains and 3 canine strains, were isolated from rectal swab samples by inoculating monolayer FK81 cells and performing a plaque purification assay. The viral and hemagglutination titers of these 41 FPV strains were 10^4.33^~10^6.33^ TCID_50_/0.1 mL and 7.0 log_2_~9.7 log_2_, respectively. Based on the complete VP2 gene, the nucleotide homology of these FPV strains was 98.91~100%, and the homology with 24 reference FPV strains from different countries and hosts was 98.85~100%. The phylogenetic analysis revealed that 41 FPV strains were more closely related to the FPV strains of Asian origin (Asian FPV strain group) than those of European and American origin (European and American FPV strain group). Furthermore, 12 mutation sites of the VP2 protein were found in these FPV strains, of which 91 and 232 amino acid sites were previously reported. Moreover, the 91 amino acid site was found to be a positive selection site with the highest dN/dS value in the selection pressure analysis. Importantly, 35 FPV strains with 91S substitution in the VP2 protein (FPV-VP2-91S strains) had formed obvious evolutionary branches in the Asian FPV strain group. The analysis of all available VP2 protein sequences of Chinese FPV strains in the GenBank database showed that the occurrence rate of FPV-VP2-91S strains had been increasing from 15.63% to 100% during 2017~2024, indicating that the FPV-VP2-91S substitution in the VP2 protein was a noteworthy molecular characteristic of the dominant FPV strains in China. These results contribute to a better understanding of their genetic evolution and renew the knowledge of FPV molecular epidemiology.

## 1. Introduction

Feline panleukopenia (FP) is caused by the feline panleukopenia virus (FPV) and is characterized by an acute, highly fatal infectious disease [1,2]. FPV is a member of the *Parvoviridae* family, with a single-stranded linear DNA, non-enveloped, icosahedral structure, and a diameter of 20–24 nm [3]. FPV contains two open reading frames, with the first ORF primarily encoding the large non-structural protein NS1. NS2, on the other hand, shares an 87-residue N-terminal domain with NS1, followed by an additional 78 amino acid residues resulting from alternative splicing [1,4]. The second ORF encodes two structural proteins: VP1 and VP2. VP1, which is also encoded by alternative splicing, includes the full sequence of VP2 and possesses a unique 143-residue N-terminal sequence that impacts the nuclear transport of capsids and the efficient infection of cells [4]. Among them, the VP2 protein is the main structural protein, accounting for 90% of all structural proteins and can affect the antigenicity, pathogenicity and host range of FPV infection [5,6]. It can self-assemble into virus-like particles and induce the production of neutralizing antibodies. Moreover, the VP2 protein plays an important role in the process of virus infection and receptor binding. Currently, the hosts of FPV infection include cats, dogs [7,8,9,10], cheetahs [11], foxes [12], leopards [13], lions [14], monkeys [15], pandas [16,17], tigers [18,19], and minks [20].

In 1983, FPV infections were first described in China, posing a significant threat to the health of local cats [21]. Currently, the prevention and control of FP are based on vaccination, and many parts of the world continue to use the 1960s-era vaccine. However, FPV has continuously mutated and evolved over time in China. Molecular surveillance carried out between 1983 and 2023 showed that FPV appeared in most areas of China [22,23,24,25,26]. However, there has been no systematic analysis of the evolutionary dynamics of FPV in East China recently. In this study, we performed isolation, genetic evolution, molecular characterization and epidemiological analysis of FPV strains in China from 2019 to 2024. We identified and analyzed the homology and phylogenetic evolution of the VP2 gene, counted the key amino acid sites in the VP2 protein and understood the dominant trend of FPV strains in China. This study enriches the strain information of FPV in China, increases the data on FPV-VP2-91S in China, and provides a basis for the molecular epidemiology of FPV.

## 2. Materials and Methods

### 2.1. Clinical Sample Collection and Virus Isolation

Feces or anal swabs (*n* = 122) were collected from sick cats and dogs suspected of disease, with symptoms including depression, loss of appetite, high temperature, vomiting, diarrhea, dehydration, and hematochezia. These samples were collected from the Veterinary Diagnostic Testing Center of Jiangsu Academy of Agricultural Sciences in Jiangsu province (*n* = 35; Nanjing, Yangzhou, Lianyungang, Nantong, Suzhou) and pet hospitals in Zhejiang province (*n* = 44,;Hangzhou, Haining), Henan province (*n* = 28; Zhengzhou) and Shanghai city (*n* = 15) from 2019 to 2024. All samples were tested using the Feline Panleukopenia colloidal gold test kit (FPV Ag, Hangzhou Evegen BIOTECH Co., Ltd., Hangzhou, China), and then FPV was detected through PCR assay as described previously [27]. Sampling and data publication were approved by the pet owners (Table S1).

To isolate pathogens, the swabs were diluted with DMEM medium (Thermo Fisher Scientific, San Jose, CA, USA) and then centrifuged. After centrifugation, the supernatant was collected and filtered through 0.22 μm microporous filter. The filtered supernatant was inoculated into monolayer cat kidney F81 cells (provided from the Shanghai Institute of Biochemistry and Cell Biology, Chinese Academy of Sciences) by the simultaneous inoculation method and cultured in DMEM medium containing 3% Fetal Bovine Serum (FBS, Thermo Fisher Scientific, San Jose, CA, USA) at 37 °C in a 5% CO_2_-humidified incubator. The supernatant was collected, frozen and thawed three times, and stored at −80 °C until use. All isolates were purified by plaque purification assay and repeated three times.

### 2.2. Viral and Hemagglutination Titers Determination

For the calculation of TCID_50_, 10-fold serial dilutions (10^−1^ to 10^−8^) were prepared from samples using sterile PBS. An amount of 100 μL from each dilution was inoculated into 96-well culture plates of monolayer F81 cells at 37 °C. After 1 h of incubation, the fresh medium was replaced, then cultured and examined daily for 5~7 d. The TCID_50_ was calculated by the Reed–Muench method as described previously [27].

The hemagglutination (HA) titer was determined by serially diluting FPV at 2-fold increments in 50 μL in a 96-well plate. To each FPV dilution, 50 μL of 1% pig red blood cell (RBC, contains 0.5% rabbit serum) working solution was added. The plates were incubated at 4 °C for 1 h before examination.

### 2.3. PCR and Sequence

Viral DNA was extracted following the instructions of the EasyPure^®^ Viral DNA Kit (Vazyme Biotech Co., Ltd., Nanjing, China). The primers were designed according to the complete ORF2 sequence of the reference FPV strain and were used to amplify the full length of VP2 gene. The primers (FPV-VP2F, 5′-CAGGACTTGTGCCTCCAG-3′, and FPV-VP2R, 5′-GCTGAGGTTGGTTATAGTGCAC-3′) were designed at the 2136~2153 position and 4467~4488 position of ORF2 and synthesized (General Biological System Co., Ltd., Chuzhou, China), which was expected to amplify a fragment of 2352 bp. The reaction conditions were as follows: 94 °C for 5 min, then 35 cycles of 94 °C for 30 s, 57 °C for 30 s, 72 °C for 2 min, followed by a final 72 °C for 10 min. The PCR products were recovered after analysis by 1% agarose gel electrophoresis. The recovered product was ligated with the pMD19-T vector (Takara Biomedical Technology (Beijing) Co., Ltd., Beijing, China) and transformed into competent *Escherichia coli* DH5α cells (Accurate Biology, Hunan, China) to screen for positive bacteria. At least three positive monoclonal colonies were sent to a biological company (General Biological System Co., Ltd., Chuzhou, China) for sequencing.

### 2.4. Genetic Evolution Analysis

The complete VP2 sequences were spliced by DNAstar Lasergene software (version 8.0). According to the FPV VP2 sequences (*n* = 24), CPV VP2 sequences (*n* = 11) and MEV VP2 sequences (*n* = 4) registered in the GenBank database, 39 reference strains (Table S1) were selected and aligned through BioEdit software (version 7.2). The nucleotide homology was performed by the MEGA software (version 7.0), and the phylogenetic tree of the VP2 gene was calculated using the model with the Maximum Likelihood (ML) method, with statistical analysis based on 1000 bootstraps. The deduced amino acid sequence was analyzed by BioEdit software (version 7.2), and the key amino acid site mutations were analyzed by DNAStar Lasergene software (version 11.1).

To estimate the selection pressure on the VP2 protein of FPV, the Datamonkey online software (http://www.datamonkey.org/, accessed on 29 October 2024) was used to infer the positive selection sites of the VP2 protein through calculating the dN/dS ratio.

### 2.5. Data Collection and Geographical Distribution Analysis

To further understand the dominance of FPV strains in China, the complete VP2 sequences of all Chinese FPV strains (*n* = 402) available in the GenBank database (accessed on 29 October 2024) and 41 strains in this study (total *n* = 443) were screened based on the VP2 protein. The corresponding information on the provinces or cities, collection date, and host was recorded. All the complete sequences of Chinese FPV VP2 protein in the GenBank database were selected to clarify the year, region, host and important amino acid site of separation (Table S3). The table includes the year, region, host, and important amino acid sites of separation for Chinese FPV strains.

## 3. Results

### 3.1. Isolation of FPV Strains

A total of 122 samples were identified as positive by the Feline Panleukopenia colloidal gold test kit and the PCR assay. The positive samples were successfully isolated and purified through the plaque purification test, and 41 FPV strains (38 strains from cats and 3 strains from dogs) were successfully isolated and purified (Table 1). The FPV positive samples were from Jiangsu province (Nanjing *n* = 2, Yangzhou *n* = 6), Zhejiang province (Hangzhou *n* = 7, Haining *n* = 3), Henan province (Zhengzhou *n* = 18) and Shanghai (*n* = 5).

### 3.2. Determination of Viral and HA Titers

**Figure 1:**
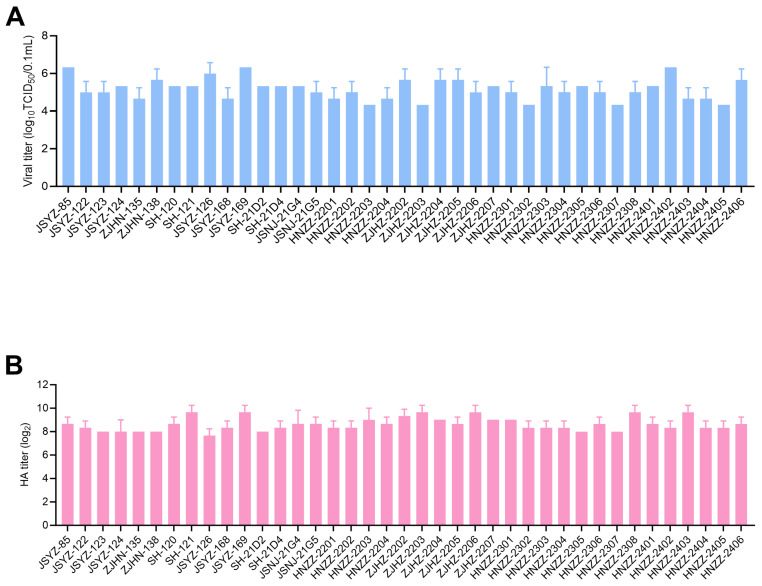
Determination of viral and hemagglutination titers of 41 FPV strains in this study. (**A**) Viral titers of 41 FPV strains in this study. (**B**) Hemagglutination titers of 41 FPV strains in this study.

The viral titers of 41 FPV strains ranged from 10^4.33^ TCID_50_/0.1 mL to 10^6.33^ TCID_50_/0.1 mL. Among them, JSYZ-85, JSYZ-169 and HNZZ-2402 were the highest titer of 10^6.33^ TCID_50_/0.1 mL. The HA titers of these FPV strains ranged from 7.0 log_2_ to 9.7 log_2_. SH-121, JYSY-169, ZJHZ-2203, ZJHZ-2206, HNZZ-2308 and HNZZ-2403 had the highest titer of 9.7 log_2_ (Figure 1).

### 3.3. Genetic Distance Analysis

The nucleotide homology analysis showed that the homology between the 41 isolates was 98.91~100%, and the similarity of 41 isolates and FPV vaccine strain was 99.02~99.54% (Figure S1).

The homology of the isolates in this study with all FPV reference strains was 98.85~100%, that of the Asian FPV strains was 98.96~100%, and that of the European and American FPV reference strains was 98.85~99.77%. The homology between 38 feline FPV strains and 3 canine FPV strains was 99.25~100%. The homology with the MEV reference strains was 98.67~99.54%, and the homology with the CPV reference strains was 97.30~98.96% (Figure S1).

### 3.4. Key Amino Acid Sites Analysis

Twelve mutation sites were found in the VP2 protein of these FPV strains, of which 91 and 232 amino acid sites were previously reported and marked in blue in Table 2. A total of 35 FPV strains were found with 91 (Ala→Ser, A91S) mutation, accounting for 85.37% (35/41), including SH-21D2, SH-21D4, SH-21G4, SH-21G5, HNZZ-2201, HNZZ-2202, HNZZ-2203, HNZZ-2204, ZJHZ-2202, ZJHZ-2203, ZJHZ-2204, ZJHZ-2207, HNZZ-2301, HNZZ-2302, HNZZ-2303, HNZZ-2304, HNZZ-2305, HNZZ-2306, HNZZ-2307, HNZZ-2308, HNZZ-2401, HNZZ-2402, HNZZ-2403, HNZZ-2404, HNZZ-2405, HNZZ-2406, JSYZ-85, ZJHN-135, ZJHN-138, SH-120, JSYZ-123, JSYZ-124, JSYZ-126, JSYZ-168, and JSYZ-169 (32 feline FPV and 3 canine FPV strains), and the V232G mutation was found in the SH-21G4 strain. In addition, 13 FPV strains, including SH-21D2 (G196D), SH-21D4 (V59A and P410S), SH-21G5 (T278A), HNZZ-2201/HNZZ-2202/HNZZ-2203 (D305Y), ZJHZ-2202 (R67G, P288L and T425S), ZJHZ-2203 (A176T, W214G), ZJHZ-2204/ZJHZ-2205/HNZZ-2306 (S35W), ZJHZ-2207 (Q280R) and HNZZ-2405 (R67G, P288L and T425S), had 12 novel mutation sites, as seen marked in red in Table 2. Compared to reference CPV strains, all isolates had 7 amino acid sites (80, 93, 103, 232, 323, 564, 568) on the VP2 protein that were significantly different from CPV virulent strains, highlighted in green in Table 2. These amino acid sites are also common key amino acid sites in the evolution process from FPV to CPV.

**Figure 2:**
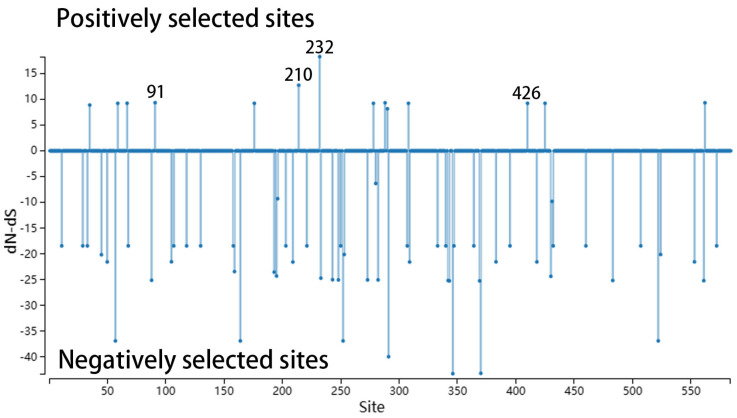
Prediction of positive selection sites of VP2 protein.

The selection pressure analysis of all Chinese FPV strains in GenBank showed that there were positive mutations at sites 38, 91, 232, 300 and 323 in the FPV sequences, and the dN/dS value at site 91 was the highest (Figure 2). Moreover, the A91S mutation site was highly frequent in 35 strains in this study. Therefore, 91 site in the VP2 protein might be a potentially important site in FPV evolution.

The selection pressure analysis of the isolates and reference strains showed that the new variants of amino acid sites 91, 210, 232 and 426 in the FPV sequence were identified at the positive selection site using the default significance level of 0.1.

### 3.5. Phylogenetic Analysis

**Figure 3:**
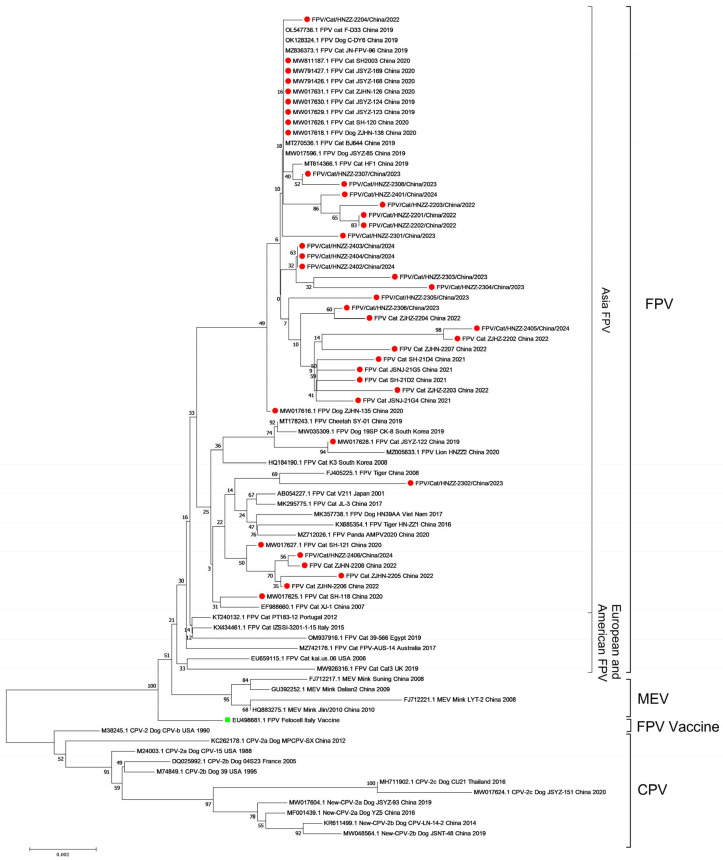
Phylogenetic analysis of the VP2 region of FPV detected in this study (2019–2024) and 39 reference FPV strains. A phylogenetic tree was constructed based on the complete VP2 gene sequences using the ML method with 1000 bootstrap replicates in the MEGA 7.0 software. Note: The FPV strains in this study are labeled with a red solid circle ( ● ). The FPV vaccine strain in this study is labeled with a green solid square (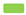).

The phylogenetic analysis based on VP2 sequences showed that the 41 FPV strains were more closely related to the FPV strains of Asian origin than the strains of European or American origin (Figure 3). At present, Asian FPV strains have become the dominant strain in China.

### 3.6. FPV Data and Geographical Distribution Analysis

**Figure 4:**
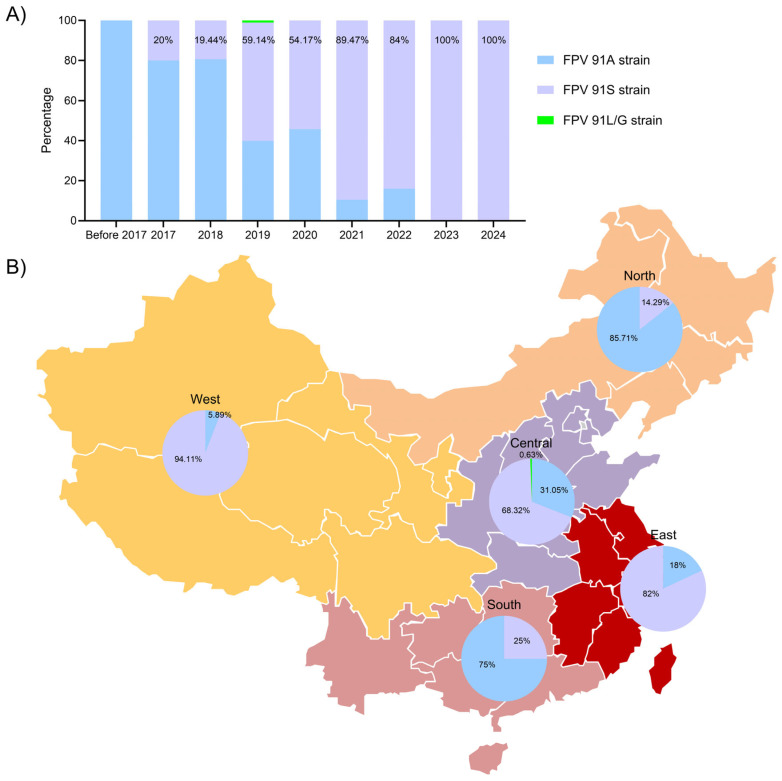
Epidemiology analysis of 443 FPV strains in China (1986–2024). (**A**) Percentage of FPV 91S variant in FPV per year. (**B**) The proportion of FPV strains with A, S, or other (L/G) at the 91 site in the five major regions of China.

Among the available 443 FPV sequences (1986~2024), the FPV-VP2-91S strain (Accession no. DQ099431.1) was first isolated in 2005, and the occurrence rate of FPV-VP2-91S strains was 1.67% (1/59) before 2017 (Figure 4). Remarkably, from 2017 to 2024, the percentages of FPV-VP2-91S strains were 15.63%, 20.37%, 57.76%, 63.63%, 71.43%, 74.29%, 57.14% and 100% (Figure 4), respectively. There were 443 FPV strains distributed in the five regions of China, consisting of North (*n* = 152), East (*n* = 106), South (*n* = 7), West (*n* = 46) and Central (*n* = 132) (Table S3). As shown in Figure 4, the percentages of FPV-VP2-91S strains in four regions (Central, East, North and West) were 65.15%, 76.42%, 24.34% and 46.65%, respectively. However, the FPV-VP2-91S strain did not appear in South China (Figure 4). The proportion of A at the 91 site was 74.34% in North China, 100% in South China, 23.58% in East China, 54.35% in West China and 34.09% in Central China. Additionally, there were a few numbers of FPV-VP2-91L/G strains that appeared in Central (0.76%) and North China (1.32%) (Figure 4). These results indicate that the FPV strains with VP2-91S characteristics are stable in Central, East and West China, and 91S has become an important molecular feature of Chinese epidemic strains in recent years.

## 4. Discussion

In China, the main vaccine used to prevent FPV is the Feline Rhinotracheitis–Calici–Panleukopenia (Elanco US Inc., Greenfield, IN, USA) inactivated virus, which is derived from the Felocell vaccine strain. However, there is a slightly lower nucleotide homology in the VP2 gene between the 41 FPV isolates circulating in China compared to the 23 reference FPV strains, including the Felocell vaccine strain. This minor genetic difference in antigenicity between the circulating strains and the vaccine strain might result in vaccine immune failure. Additionally, some cats may be infected with other pathogens such as feline leukemia virus [26], which can weaken the immune system and lead to cross-infections, with clinical symptoms primarily caused by FPV. Studies have also shown that even vaccinated cats could still get infected with FPV, possibly due to variations in maternally derived antibody (MDA) levels [28]. Cats with higher MDA levels may not respond well to the initial vaccinations, leaving them vulnerable to infection for a longer period. This could explain why some vaccinated cats still become infected with FPV. Additionally, when cats are under physiological stress, their immune system could be weakened, making them more prone to infections like FPV.

Both FPV and CPV belong to the family of *Parvoviridae*, and they might have originated from a common ancestor based on genetic evolution [5,6]. However, mutations in certain amino acid sites could alter the binding ability of FPV or CPV to host transferrin receptor (TfR), leading to changes in host range [8,28]. In this study, the genetic evolution of feline and canine FPV strains was highly homologous, similar to the results of Palermo, L.M et al. [28]. In recent years, FPV infection in dogs has been reported in some countries such as Pakistan [7], Vietnam [9], Thailand [29], Italy, Egypt [8] and China [10]. Some studies on canine FPV have shown that FPV has the ability to spread to dogs [7]. Additionally, keeping dogs and cats together as a standard practice might lead to an increased likelihood of dogs becoming infected with FPV. Based on the clinical symptoms we gathered (Table 1), it is our belief that the occurrence of FPV infection in dogs is not random. With an increasing number of reported cases, it is crucial for us to take notice.

The VP2 protein, being the primary component of FPV capsid protein, plays a crucial role in determining the specificity of TfR binding in feline and canine hosts, as well as influencing the virus’ host range and the susceptibility of host cells. The tertiary structure of the VP2 protein is primarily composed of five loop structures, including Loop1 (amino acid residues 50–100), Loop2 (amino acid residues 200–250), Loop3 (amino acid residues 350–400), Loop4 (amino acid residues 400–450), and FlexibleLoop5 (amino acid residues 350–400). Among these, Loop1, Loop2, and Loop4 together form the top structure of the three-fold spike of the VP2 protein, known as epitope A, while Loop3 represents epitope B located at the VP2 protein shoulder [30].

The amino acid sites 91 and 232 were reported in previous research [31,32,33]. The proportion of FPV-VP2-91S in this study was 85.37% (35/41). Although previous studies have shown that FPV is conserved in genetic evolution [3], this study shows that FPV is evolving. The VP2 protein of FPV is characterized by five loop structures, with the random loop structure located in the protuberance region of the VP2 capsid coil, containing epitopes that are essential for transferrin receptor binding and infection [34,35]. The 91 site of VP2 is located in the Loop1 structure, which is in close proximity to the 93 site. Residue 93 is a critical determinant that affects TfR binding, host range, and reactivity with specific antibodies in canines and felines [36,37,38,39,40,41], and is one of the key sites that control the difference between FPV and CPV-2 [28,35,42,43]. Furthermore, the study by Xi Chen et al [22]. showed that the A91S mutation in FPV VP2 protein extended the random coil of amino acid residues from 92–95 to 91–95 in the Loop1 domain through 3D structure prediction [22]. Therefore, it is speculated that the mutation of the 91 site in VP2 protein from A to S may affect the structure of the 93 site in Loop1, which could impact the interaction with the TfR of feline and canine [44,45]. This mutation may impact the self-assembly of VP2 protein and its ability to infect hosts.

The homology of CPV-2 and FPV is very high [5,38,46], and both of them achieve virus invasion by binding to TfR receptors [28,30,47,48]. Although it was previously believed that CPV could infect cats [49], FPV could not infect dogs [50,51]. However, some recent studies have shown that FPV could be successfully isolated from diseased dogs [10]. This might also be related to the 91-site mutation, which affects the structural region [32], thus affecting the recognition of FPV and canine TfR receptor. In summary, it is important to be aware of the increasing number of cases of FPV infection in dogs in urban areas, possibly due to the growing population of dogs and cats living together.

Steinel et al. found that only six key amino acids on VP2 (80, 93, 103, 323, 564, and 568) need to change for FPV to evolve into CPV-2 [52]. Specifically, amino acid residues at positions 80 (located in Loop1), 93 (located in Loop1), 232 (located in Loop2), and 323 (located in Loop3) may all affect the binding ability of VP2 protein to TFR from different host species. The 36 of the 41 strains had 14 mutation sites between them, involving S35W, V59A, R67G, A91S, A176T, G196D, W214G, V232G, T278A, Q280R, P288L, D305Y, P410S and T425S. Interestingly, these identified amino acid substitutions have occurred in different loop structures of the VP2 protein. For example, the 214th position on Loop 2, and the 410th and 425th positions on Loop 4, might alter epitope A formed by Loop1, Loop 2, and Loop 4, collectively, thereby affecting the spatial configuration of VP2 protein. Additionally, Allison AB et al. reported changes at VP2 position 299 or 301 in association with CPV VP2 position 300 mutants [33], suggesting that substitutions at amino acid positions near loop structures might also impact the structure and function of the VP2 protein. Similarly, our study found substitutions at amino acid positions near loop structures. Although current molecular epidemiological data are limited, and these substitutions at these positions are occurring for the first time, further research is needed to elucidate their impact on the function of FPV VP2 protein.

Phylogenetic analysis showed that FPV strains could be categorized into two groups: the Asian FPV group and the European and American FPV group, which aligns with previous reports [27]. All the 41 FPV strains in this study belonged to the Asian FPV group and displayed a distant relationship with the vaccine strain. The FPV-VP2-91S strains formed obvious evolutionary branches, indicating that the 91Ser substitution in the VP2 protein is a significant molecular characteristic of the dominant FPV strains and could serve as a molecular typing marker.

Finally, all the available Chinese FPV strains on NCBI were analyzed, and it was found that the first FPV-VP2-91S strain was discovered in Jilin Province, China in 2005. Since 2017, the A91S strain has shown explosive growth in China. From 2017 to 2024, the percentage of FPV-VP2-91S strains has been increasing. Among the five major regions of China, the proportion of A91S strains is the highest, showing that the FPV-VP2-91S strains are very serious in some parts of China. This finding aligns with the conclusions drawn by Chen X [22] and Xue H [33] et al. in recent studies.

## 5. Conclusions

In summary, this study enhanced the data on FPV in China and revealed that FPV-VP2-91S was the dominant type in the country. By incorporating data from all FPV strains in China, the results showed that the FPV-VP2-91S strain had become the dominant strain in China and might become the dominant epidemic strain. The results of this study will help to understand the distribution of FPV in China and looks forward to providing a theoretical basis for the prevention and control of FPV.

## Figures and Tables

**Table 1:** Basic information of 41 FPV isolates detected in this study (2019–2024).

Sample	Year	Origin	Host	Clinical Feature	Age	Vaccination (N/1 ^a^/2 ^b^/3 ^c^)	GenBank Accession No.
JSYZ-85	2019	China/Jiangsu	Dog	High fever, diarrhea, dehydration	5 months	2 ^d^	MW017596.1
JSYZ-122	2019	China/Jiangsu	Cat	High fever, diarrhea	4 months	1	MW017628.1
JSYZ-123	2019	China/Jiangsu	Cat	High fever, diarrhea, dehydration	1 months	N	MW017629.1
JSYZ-124	2019	China/Jiangsu	Cat	High fever, vomit, diarrhea, dehydration	2 months	N	MW017630.1
ZJHN-135	2020	China/Zhejiang	Dog	High fever, diarrhea, dehydration	3 months	1 ^d^	MW017616.1
ZJHN-138	2020	China/Zhejiang	Dog	High fever, vomit	1 months	N	MW017618.1
SH-118	2020	China/Shanghai	Cat	High fever	4 months	2	MW017625.1
SH-120	2020	China/Shanghai	Cat	High fever, vomit, diarrhea	3 months	N	MW017626.1
SH-121	2020	China/Shanghai	Cat	High fever, diarrhea	3 months	N	MW017627.1
ZJHN-126	2020	China/Zhejiang	Cat	High fever, vomit, diarrhea, dehydration	3 months	1	MW017631.1
JSYZ-168	2020	China/Jiangsu	Cat	High fever, vomit, diarrhea	4 months	1	MW791426.1
JSYZ-169	2020	China/Jiangsu	Cat	High fever, diarrhea, dehydration	3 months	1	MW791427.1
SH-21D2	2021	China/Shanghai	Cat	High fever, vomit	4 months	2	OP796706
SH-21D4	2021	China/Shanghai	Cat	High fever	4 months	1	OP796707
JSNJ-21G4	2021	China/Jiangsu	Cat	High fever, vomit, diarrhea	3 months	N	OP796708
JSNJ-21G5	2021	China/Jiangsu	Cat	High fever, vomit, diarrhea	6 months	3	OP796709
HNZZ-2201	2022	China/Henan	Cat	High fever, diarrhea, dehydration	3 months	N	PQ560975
HNZZ-2202	2022	China/Henan	Cat	High fever, diarrhea	6 months	2	PQ560976
HNZZ-2203	2022	China/Henan	Cat	High fever, vomit, diarrhea	3 months	1	PQ560977
HNZZ-2204	2022	China/Henan	Cat	High fever, diarrhea	5 months	2	PQ560978
ZJHZ-2202	2022	China/Zhejiang	Cat	High fever, diarrhea	5 months	N	OP796710
ZJHZ-2203	2022	China/Zhejiang	Cat	High fever, vomit, diarrhea	5 months	2	OP796711
ZJHZ-2204	2022	China/Zhejiang	Cat	High fever, diarrhea, dehydration	4 months	N	OP796712
ZJHZ-2205	2022	China/Zhejiang	Cat	High fever, diarrhea, dehydration	8 months	3	OP796713
ZJHZ-2206	2022	China/Zhejiang	Cat	High fever, vomit, diarrhea, dehydration	6 months	2	OP796714
ZJHZ-2207	2022	China/Zhejiang	Cat	High fever, diarrhea, dehydration	6 months	N	OP796715
ZJHZ-2208	2022	China/Zhejiang	Cat	High fever, diarrhea	11 months	3	OP796716
HNZZ-2301	2023	China/Henan	Cat	High fever	4 months	2	PQ560979
HNZZ-2302	2023	China/Henan	Cat	High fever, vomit, diarrhea	3 months	N	PQ560980
HNZZ-2303	2023	China/Henan	Cat	High fever, diarrhea	3 months	N	PQ560981
HNZZ-2304	2023	China/Henan	Cat	High fever, vomit, diarrhea, dehydration	3 months	1	PQ560982
HNZZ-2305	2023	China/Henan	Cat	High fever, vomit, diarrhea	4 months	1	PQ560983
HNZZ-2306	2023	China/Henan	Cat	High fever	3 months	N	PQ560984
HNZZ-2307	2023	China/Henan	Cat	High fever	3 months	N	PQ560985
HNZZ-2308	2023	China/Henan	Cat	High fever, vomit, diarrhea, dehydration	6 months	2	PQ560986
HNZZ-2401	2024	China/Henan	Cat	High fever, diarrhea	4 months	1	PQ560987
HNZZ-2402	2024	China/Henan	Cat	High fever, diarrhea, dehydration	1 months	N	PQ560988
HNZZ-2403	2024	China/Henan	Cat	High fever, vomit, diarrhea, dehydration	2 months	N	PQ560989
HNZZ-2404	2024	China/Henan	Cat	High fever, diarrhea, dehydration	2 months	N	PQ560990
HNZZ-2405	2024	China/Henan	Cat	High fever	11 months	3	PQ560991
HNZZ-2406	2024	China/Henan	Cat	High fever, vomit, diarrhea	4 months	2	PQ560992

Note: ^a^ Pets were vaccinated once with the Feline Rhinotracheitis–Calici-Panleukopenia vaccine. ^b^ Pets were vaccinated twice with the Feline Rhinotracheitis–Calici-Panleukopenia vaccine. ^c^ Pets were vaccinated three times with the Feline Rhinotracheitis–Calici–Panleukopenia vaccine. ^d^ Dogs were vaccinated with the Canine Distemper–Adenovirus Type2–Coronavirus–Parainfluenza–Parvovirus vaccine, modified live and killed virus, and *Leptospira Canicola-Icterohaemorrhagiae* bacterin vaccine.

**Table 2:** Amino acid mutations in the VP2 proteins of FPVs, MEVs and CPVs.

Strain	Virus or Genotype	35	59	67	80	91	93	101	103	176	196	214	232	278	280	288	305	323	410	425	426	564	568
SH-21D2	FPV	S	V	R	K	S	K	T	V	A	D	W	V	T	Q	P	D	D	P	T	N	N	A
SH-21D4	FPV	S	A	R	K	S	K	T	V	A	G	W	V	T	Q	P	D	D	S	T	N	N	A
JSNJ-21G4	FPV	S	V	R	K	S	K	T	V	A	G	W	G	T	Q	P	D	D	P	T	N	N	A
JSNJ-21G5	FPV	S	V	R	K	S	K	T	V	A	G	W	V	A	Q	P	D	D	P	T	N	N	A
HNZZ-2201	FPV	S	V	R	K	S	K	T	V	A	G	W	V	T	Q	P	Y	D	P	T	N	N	A
HNZZ-2202	FPV	S	V	R	K	S	K	T	V	A	G	W	V	T	Q	P	Y	D	P	T	N	N	A
HNZZ-2203	FPV	S	V	R	K	S	K	T	V	A	G	W	V	T	Q	P	Y	D	P	T	N	N	A
HNZZ-2204	FPV	S	V	R	K	S	K	T	V	A	G	W	V	T	Q	P	D	D	P	T	N	N	A
ZJHZ-2202	FPV	S	V	G	K	S	K	T	V	A	G	W	V	T	Q	L	D	D	P	S	N	N	A
ZJHZ-2203	FPV	S	V	R	K	S	K	T	V	T	G	G	V	T	Q	P	D	D	P	T	N	N	A
ZJHZ-2204	FPV	W	V	R	K	S	K	T	V	A	G	W	V	T	Q	P	D	D	P	T	N	N	A
ZJHZ-2205	FPV	W	V	R	K	A	K	T	V	A	G	W	V	T	Q	P	D	D	P	T	N	N	A
ZJHZ-2206	FPV	S	V	R	K	A	K	T	V	A	G	W	V	T	Q	P	D	D	P	T	N	N	A
ZJHZ-2207	FPV	S	V	R	K	S	K	T	V	A	G	W	V	T	R	P	D	D	P	T	N	N	A
ZJHZ-2208	FPV	S	V	R	K	A	K	T	V	A	G	W	V	T	Q	P	D	D	P	T	N	N	A
HNZZ-2301	FPV	S	V	R	K	S	K	T	V	A	G	W	G	T	Q	P	D	D	P	T	N	N	A
HNZZ-2302	FPV	S	V	R	K	S	K	I	V	A	G	W	G	T	Q	P	D	D	P	T	N	N	A
HNZZ-2303	FPV	S	V	R	K	S	K	T	V	A	G	W	V	T	Q	P	D	D	P	T	N	N	A
HNZZ-2304	FPV	S	V	R	K	S	K	T	V	A	G	W	V	T	Q	P	D	D	P	T	N	N	A
HNZZ-2305	FPV	S	V	R	K	S	K	T	V	A	G	W	V	T	Q	P	D	D	P	T	N	N	A
HNZZ-2306	FPV	W	V	R	K	S	K	T	V	A	G	W	V	T	Q	P	D	D	P	T	N	N	A
HNZZ-2307	FPV	S	V	R	K	S	K	T	V	A	G	W	V	T	Q	P	D	D	P	T	N	N	A
HNZZ-2308	FPV	S	V	R	K	S	K	T	V	A	G	W	V	T	Q	P	D	D	P	T	N	N	A
HNZZ-2401	FPV	S	V	R	K	S	K	T	V	A	G	W	V	T	Q	P	D	D	P	T	N	N	A
HNZZ-2402	FPV	S	V	R	K	S	K	T	V	A	G	W	V	T	Q	P	D	D	P	T	N	N	A
HNZZ-2403	FPV	S	V	R	K	S	K	T	V	A	G	W	V	T	Q	P	D	D	P	T	N	N	A
HNZZ-2404	FPV	S	V	R	K	S	K	T	V	A	G	W	V	T	Q	P	D	D	P	T	N	N	A
HNZZ-2405	FPV	S	V	G	K	S	K	T	V	A	G	W	V	T	Q	L	D	D	P	S	N	N	A
HNZZ-2406	FPV	S	V	R	K	S	K	T	V	A	G	W	V	T	Q	P	D	D	P	T	N	N	A
JSYZ-85	FPV	S	V	R	K	S	K	T	V	A	G	W	V	T	Q	P	D	D	P	T	N	N	A
ZJHN-135	FPV	S	V	R	K	S	K	T	V	A	G	W	V	T	Q	P	D	D	P	T	N	N	A
ZJHN-138	FPV	S	V	R	K	S	K	T	V	A	G	W	V	T	Q	P	D	D	P	T	N	N	A
SH-118	FPV	S	V	R	K	A	K	T	V	A	G	W	V	T	Q	P	D	D	P	T	N	N	A
SH-120	FPV	S	V	R	K	S	K	T	V	A	G	W	V	T	Q	P	D	D	P	T	N	N	A
SH-121	FPV	S	V	R	K	A	K	T	V	A	G	W	V	T	Q	P	D	D	P	T	N	N	A
JSYZ-122	FPV	S	V	R	K	A	K	T	V	A	G	W	V	T	Q	P	D	D	P	T	N	N	A
JSYZ-123	FPV	S	V	R	K	S	K	T	V	A	G	W	V	T	Q	P	D	D	P	T	N	N	A
JSYZ-124	FPV	S	V	R	K	S	K	T	V	A	G	W	V	T	Q	P	D	D	P	T	N	N	A
ZJHN-126	FPV	S	V	R	K	S	K	T	V	A	G	W	V	T	Q	P	D	D	P	T	N	N	A
JSYZ-168	FPV	S	V	R	K	S	K	T	V	A	G	W	V	T	Q	P	D	D	P	T	N	N	A
JSYZ-169	FPV	S	V	R	K	S	K	T	V	A	G	W	V	T	Q	P	D	D	P	T	N	N	A
Felocell (Vaccine)	FPV	S	V	R	K	A	K	T	V	A	G	W	I	T	Q	P	D	D	P	T	N	N	A
XJ-1	FPV	S	V	R	K	A	K	T	V	A	G	W	V	T	Q	P	D	D	P	T	N	N	A
JL-3	FPV	S	V	R	K	A	K	T	V	A	G	W	V	T	Q	P	D	D	P	T	N	N	A
BJ644	FPV	S	V	R	K	S	K	T	V	A	G	W	V	T	Q	P	D	D	P	T	N	N	A
HF1	FPV	S	V	R	K	S	K	T	V	A	G	W	V	T	Q	P	D	D	P	T	N	N	A
F-D33	FPV	S	V	R	K	S	K	T	V	A	G	W	V	T	Q	P	D	D	P	T	N	N	A
JN-FPV-96	FPV	S	V	R	K	S	K	T	V	A	G	W	V	T	Q	P	D	D	P	T	N	N	A
SH2003	FPV	S	V	R	K	S	K	T	V	A	G	W	V	T	Q	P	D	D	P	T	N	N	A
V211	FPV	S	V	R	K	A	K	T	V	A	G	W	V	T	Q	P	D	D	P	T	N	N	A
K3	FPV	S	V	R	K	A	K	T	V	A	G	W	V	T	Q	P	D	D	P	T	N	N	A
C-DY6	FPV	S	V	R	K	S	K	T	V	A	G	W	V	T	Q	P	D	D	P	T	N	N	A
HN39AA	FPV	S	V	R	K	A	K	T	V	A	G	W	V	T	Q	P	D	D	P	T	N	N	A
19SP_CK-8	FPV	S	V	R	K	A	K	T	V	A	G	W	V	T	Q	P	D	D	P	T	N	N	A
Tiger	FPV	S	V	R	K	A	K	T	V	A	G	W	V	T	Q	P	D	D	P	T	N	N	A
HN-ZZ1	FPV	S	V	R	K	A	K	T	V	A	G	W	V	T	Q	P	D	D	P	T	N	N	A
AMPV2020	FPV	S	V	R	K	A	K	T	V	A	G	W	V	T	Q	P	D	D	P	T	N	N	A
SY-01/2019	FPV	S	V	R	K	A	K	T	V	A	G	W	V	T	Q	P	D	D	P	T	N	N	A
HNZZ2	FPV	S	V	R	K	A	K	T	V	A	G	W	V	T	Q	P	D	D	P	T	N	N	A
Cat 3	FPV	S	V	R	K	A	K	T	V	A	G	W	V	T	Q	P	D	D	P	T	N	N	A
kai.us.06	FPV	S	V	R	K	A	K	T	V	A	G	W	V	T	Q	P	D	D	P	T	N	N	A
AUS-14	FPV	S	V	R	K	A	K	T	V	A	G	W	V	T	Q	P	D	D	P	T	N	N	A
39–566	FPV	S	V	R	K	A	K	T	V	A	G	W	V	T	Q	P	D	D	P	T	N	N	A
PT183/12	FPV	S	V	R	K	A	K	T	V	A	G	W	V	T	Q	P	D	D	P	T	N	N	A
IZSSI_3201_1_15	FPV	S	V	R	K	A	K	T	V	A	G	W	V	T	Q	P	D	D	P	T	N	N	A
Suning	MEV	S	V	R	K	A	K	T	V	A	G	W	V	T	Q	P	D	D	P	T	N	N	A
LYT-2	MEV	S	V	R	K	A	K	T	V	A	G	W	V	T	Q	P	D	D	P	T	K	N	A
Dalian2	MEV	S	V	R	K	A	K	T	V	A	G	W	V	T	Q	P	D	D	P	T	N	N	A
Jlin/2010	MEV	S	V	R	K	A	K	T	V	A	G	W	V	T	Q	P	D	D	P	T	N	N	A
CPV-b	CPV-2	S	V	R	R	A	N	I	A	A	G	W	I	T	Q	P	D	N	P	T	N	S	G
CPV-15	CPV-2a	S	V	R	R	A	N	T	A	A	G	W	I	T	Q	P	Y	N	P	T	N	S	G
MPCPV-SX	CPV-2a	S	V	R	R	V	N	T	A	A	G	W	I	T	Q	P	D	N	P	T	N	S	G
39	CPV-2b	S	V	R	R	A	N	T	A	A	G	W	I	T	Q	P	Y	N	P	T	D	S	G
04S23	CPV-2b	S	V	R	R	A	N	T	A	A	G	W	I	T	Q	P	Y	N	P	T	D	S	G
YZ5	New CPV-2a	S	V	R	R	A	N	T	A	A	G	W	I	T	Q	P	Y	N	P	T	N	S	G
JSYZ-93	New CPV-2a	S	V	R	R	A	N	T	A	A	G	W	I	T	Q	P	Y	N	P	T	N	S	G
JSNT-48	New CPV-2b	S	V	R	R	A	N	T	A	A	G	W	I	T	Q	P	Y	N	P	T	D	S	G
LN-14-2	New CPV-2b	S	V	R	R	A	N	T	A	A	G	W	I	T	Q	P	Y	N	P	T	D	S	G
CU21	CPV-2c	S	V	R	R	A	N	T	A	A	G	W	I	T	Q	P	Y	N	P	T	E	S	G
JSYZ-151	CPV-2c	S	V	R	R	A	N	T	A	A	G	W	I	T	Q	P	Y	N	P	T	E	S	G

Note: 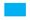 well-recognized mutations compared with vaccine FPV strain. 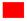 novel mutations compared with vaccine FPV strain. 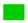 well-recognized mutations compared with reference CPV strain.

## Data Availability

The FPV strain data obtained in this study are available from NCBI (https://www.ncbi.nlm.nih.gov/, accessed on 29 October 2024), and others are presented in this study and its Supplementary Materials. The source code is available from the corresponding author upon request.

## Supplementary Materials

The following supporting information can be downloaded at: https://www.mdpi.com/article/10.3390/v16121967/s1, Table S1. Basic information and detection of 94 feces or anal swabs samples. Table S2. Basic information of 39 reference FPV, CPV and MEV strains. Table S3. Complete sequences of Chinese FPV VP2 protein in the GenBank database. Figure S1. Sequence homology.
