# Supplementary material for: Epidemiological and Molecular Investigation of Feline Panleukopenia Virus Infection in China
Source: Viruses. 2024 Dec 23;16(12):1967. doi: 10.3390/v16121967 (PMC11728606; doi:10.3390/v16121967)
Supplement: Supplementary file 1 [file viruses-16-01967-s001.zip › Table S1 122╖▌▓í┴╧╡─▒│╛░╨┼╧ó.pdf]

Table S1. Basic information and detection of 94 feces or anal swabs samples.

Sample	Year	Origin	Host	Age	Vaccination (Y/N)	Clinical feature	Ag(P/N)	PCR(P/N)	Virus isolation(P/N)
JSYZ-83	2019	China/Jiangsu	Cat	3 months	Y	High fever, diarrhea, dehydration	P	P	N
JSYZ-84	2019	China/Jiangsu	Cat	3 months	Y	High fever, vomit, diarrhea, dehydration	P	P	N
JSYZ-85	2019	China/Jiangsu	Dog	5 months	Y	High fever, diarrhea, dehydration	P	P	P
JSYZ-86	2019	China/Jiangsu	Cat	2 months	N	High fever, vomit, diarrhea, dehydration	P	P	N
JSYZ-121	2019	China/Jiangsu	Cat	3 months	Y	High fever, vomit, diarrhea, dehydration	P	P	N
JSYZ-122	2019	China/Jiangsu	Cat	4 months	Y	High fever, diarrhea	P	P	P
JSYZ-123	2019	China/Jiangsu	Cat	1 months	N	High fever, diarrhea, dehydration	P	P	P
JSYZ-124	2019	China/Jiangsu	Cat	2 months	N	High fever, vomit, diarrhea, dehydration	P	P	P
JSYZ-125	2019	China/Jiangsu	Cat	5 months	Y	High fever, vomit, diarrhea	P	P	N
JSYZ-126	2020	China/Jiangsu	Cat	3 months	Y	High fever, vomit, diarrhea, dehydration	P	P	P
JSYZ-127	2020	China/Jiangsu	Cat	5 months	Y	High fever, diarrhea	N	N	N
JSYZ-128	2020	China/Jiangsu	Cat	6 months	Y	High fever, diarrhea	N	N	N
JSYZ-129	2020	China/Jiangsu	Cat	2 months	Y	High fever, vomit, diarrhea, dehydration	P	P	N
JSYZ-130	2020	China/Jiangsu	Dog	5 months	Y	High fever	N	N	N
JSYZ-131	2021	China/Jiangsu	Cat	4 months	Y	High fever, vomit	N	N	N
JSYZ-132	2021	China/Jiangsu	Cat	4 months	Y	High fever, vomit, diarrhea, dehydration	P	P	N
JSYZ-134	2021	China/Jiangsu	Cat	4 months	Y	High fever, diarrhea, dehydration	P	P	N
JSYZ-135	2021	China/Jiangsu	Cat	2 months	N	High fever, diarrhea, dehydration	P	P	N
JSYZ-136	2021	China/Jiangsu	Cat	3 months	Y	High fever, vomit, diarrhea, dehydration	P	P	N
JSYZ-137	2022	China/Jiangsu	Cat	5 months	Y	High fever	N	N	N
JSYZ-138	2022	China/Jiangsu	Cat	2 months	Y	High fever, vomit, diarrhea, dehydration	P	P	N
JSYZ-168	2020	China/Jiangsu	Cat	4 months	Y	High fever, vomit, diarrhea	P	P	P
JSYZ-169	2020	China/Jiangsu	Cat	3 months	Y	High fever, diarrhea, dehydration	P	P	P

JSYZ-170	2020	China/Jiangsu	Cat	2 months	Y	High fever, vomit, diarrhea, dehydration	P	P	N
SH-115	2020	China/Shanghai	Cat	2 months	Y	High fever, vomit, diarrhea, dehydration	P	P	N
SH-116	2020	China/Shanghai	Cat	7 months	Y	High fever, diarrhea, dehydration	N	N	N
SH-117	2020	China/Shanghai	Cat	3 months	Y	High fever, vomit, diarrhea, dehydration	P	P	N
SH-118	2020	China/Shanghai	Cat	4 months	Y	High fever	P	P	P
SH-119	2020	China/Shanghai	Cat	2 months	N	High fever, vomit, diarrhea, dehydration	P	P	N
SH-120	2020	China/Shanghai	Cat	3 months	N	High fever, vomit, diarrhea	P	P	P
SH-121	2020	China/Shanghai	Cat	3 months	N	High fever, diarrhea	P	P	P
SH-122	2020	China/Shanghai	Cat	6 months	Y	High fever, diarrhea, dehydration	P	P	N
SH-21D1	2021	China/Shanghai	Cat	2 months	Y	High fever, vomit, diarrhea, dehydration	P	P	N
SH-21D2	2021	China/Shanghai	Cat	4 months	Y	High fever, vomit	P	P	P
SH-21D3	2021	China/Shanghai	Cat	2 months	N	High fever, diarrhea, dehydration	P	P	N
SH-21D4	2021	China/Shanghai	Cat	4 months	Y	High fever	N	N	P
SH-21D5	2021	China/Shanghai	Cat	3 months	Y	High fever, vomit, diarrhea, dehydration	P	P	N
SH-21D6	2021	China/Shanghai	Cat	6 months	Y	High fever, vomit	N	N	N
SH-21D7	2021	China/Shanghai	Cat	6 months	Y	High fever	N	N	N
ZJHN-126	2020	China/Zhejiang	Cat	5 months	Y	High fever, diarrhea	P	P	N
ZJHN-127	2020	China/Zhejiang	Cat	2 months	Y	High fever, diarrhea, dehydration	P	P	N
ZJHN-128	2020	China/Zhejiang	Cat	3 months	Y	High fever, vomit, diarrhea, dehydration	P	P	N
ZJHN-129	2020	China/Zhejiang	Cat	4 months	Y	High fever, diarrhea, dehydration	P	P	N
ZJHN-130	2020	China/Zhejiang	Cat	6 months	Y	High fever	P	P	N
ZJHN-131	2020	China/Zhejiang	Cat	4 months	Y	High fever, vomit, diarrhea, dehydration	P	P	N
ZJHN-132	2020	China/Zhejiang	Cat	2 months	N	High fever, vomit, diarrhea	P	P	N
ZJHN-133	2020	China/Zhejiang	Cat	5 months	Y	High fever	N	N	N
ZJHN-134	2020	China/Zhejiang	Cat	5 months	Y	High fever	N	N	N
ZJHN-135	2020	China/Zhejiang	Dog	3 months	Y	High fever, diarrhea, dehydration	P	P	P

ZJHN-136	2020	China/Zhejiang	Cat	2 months	N	High fever, vomit, diarrhea, dehydration	P	P	N
ZJHN-137	2020	China/Zhejiang	Cat	6 months	Y	High fever, diarrhea	P	P	N
ZJHN-138	2020	China/Zhejiang	Dog	1 months	N	High fever, vomit	P	P	P
ZJHN-139	2020	China/Zhejiang	Cat	5 months	Y	High fever	N	N	N
ZJHN-140	2020	China/Zhejiang	Cat	6 months	Y	High fever, vomit, diarrhea, dehydration	P	P	N
ZJHN-141	2020	China/Zhejiang	Cat	6 months	Y	High fever	P	P	N
ZJHN-142	2020	China/Zhejiang	Cat	4 months	Y	High fever, diarrhea, dehydration	P	P	N
ZJHN-143	2020	China/Zhejiang	Cat	1 months	N	High fever, diarrhea, dehydration	P	P	N
ZJHN-144	2021	China/Zhejiang	Cat	3 months	Y	High fever, vomit, diarrhea, dehydration	P	P	N
ZJHN-145	2021	China/Zhejiang	Cat	5 months	Y	High fever, vomit, diarrhea	P	P	N
ZJHN-146	2021	China/Zhejiang	Cat	4 months	Y	High fever, vomit, diarrhea	P	P	N
ZJHN-147	2021	China/Zhejiang	Dog	2 months	N	High fever, vomit, diarrhea, dehydration	P	P	N
ZJHN-148	2022	China/Zhejiang	Cat	5 months	Y	High fever, vomit, diarrhea	P	P	N
ZJHN-149	2022	China/Zhejiang	Cat	4 months	Y	High fever, diarrhea, dehydration	P	P	N
ZJHN-150	2022	China/Zhejiang	Cat	4 months	Y	High fever, vomit, diarrhea, dehydration	P	P	N
ZJHN-151	2022	China/Zhejiang	Cat	4 months	Y	High fever, vomit, diarrhea, dehydration	P	P	N
ZJHN-152	2022	China/Zhejiang	Cat	6 months	N	High fever	N	N	N
ZJHZ-21F1	2021	China/Zhejiang	Cat	2 months	Y	High fever, vomit, diarrhea	P	P	N
ZJHZ-21F2	2021	China/Zhejiang	Cat	4 months	Y	High fever, vomit, diarrhea	P	P	N
ZJHZ-21F3	2021	China/Zhejiang	Cat	6 months	Y	High fever, diarrhea	N	N	N
ZJHZ-21F4	2021	China/Zhejiang	Cat	8 months	Y	High fever, vomit	N	N	N
ZJHZ-21F5	2021	China/Zhejiang	Cat	3 months	Y	High fever, vomit, diarrhea, dehydration	P	P	N
ZJHZ-21F6	2021	China/Zhejiang	Cat	2 months	Y	High fever, diarrhea, dehydration	P	P	N
ZJHZ-21F7	2021	China/Zhejiang	Cat	5 months	Y	High fever	N	N	N
ZJHZ-21F8	2021	China/Zhejiang	Cat	2 months	Y	High fever, vomit, diarrhea	P	P	N
ZJHZ-2201	2022	China/Zhejiang	Cat	3 months	Y	High fever, diarrhea	P	P	N

ZJHZ-2202	2022	China/Zhejiang	Cat	5 months	N	High fever, diarrhea	P	P	P
ZJHZ-2203	2022	China/Zhejiang	Cat	5 months	Y	High fever, vomit, diarrhea	P	P	P
ZJHZ-2204	2022	China/Zhejiang	Cat	4 months	N	High fever, diarrhea, dehydration	P	P	P
ZJHZ-2205	2022	China/Zhejiang	Cat	8 months	Y	High fever, diarrhea, dehydration	P	P	P
ZJHZ-2206	2022	China/Zhejiang	Cat	6 months	Y	High fever, vomit, diarrhea, dehydration	P	P	P
ZJHZ-2207	2022	China/Zhejiang	Cat	6 months	N	High fever, diarrhea, dehydration	P	P	P
ZJHZ-2208	2022	China/Zhejiang	Cat	11 months	Y	High fever, diarrhea	P	P	P
JSNJ-20A1	2020	China/Jiangsu	Cat	4 months	Y	High fever, diarrhea, dehydration	P	P	N
JSNJ-20A2	2020	China/Jiangsu	Cat	3 months	Y	High fever, diarrhea	P	P	N
JSNJ-21F3	2021	China/Jiangsu	Cat	2 months	N	High fever, vomit, diarrhea	P	P	N
JSNJ-21F4	2021	China/Jiangsu	Cat	4 months	Y	High fever, diarrhea, dehydration	P	P	N
JSNJ-21F5	2021	China/Jiangsu	Cat	2 months	Y	High fever, diarrhea, dehydration	P	P	N
JSNJ-21G4	2021	China/Jiangsu	Cat	3 months	N	High fever, vomit, diarrhea	P	P	P
JSNJ-21G5	2021	China/Jiangsu	Cat	6 months	Y	High fever, diarrhea	P	P	P
JSNJ-22H7	2022	China/Jiangsu	Cat	5 months	Y	High fever	N	N	N
JSNJ-22H8	2022	China/Jiangsu	Cat	5 months	Y	High fever, diarrhea	P	P	N
JSNJ-22H9	2022	China/Jiangsu	Dog	3 months	Y	High fever, diarrhea, dehydration	N	N	N
JSNJ-22I9	2022	China/Jiangsu	Cat	2 months	N	High fever, vomit, diarrhea	P	P	N
JSNJ-22I10	2022	China/Jiangsu	Cat	4 months	Y	High fever, vomit, diarrhea, dehydration	P	P	N
HNZZ-2201	2022	China/Henan	Cat	3 months	N	High fever, diarrhea, dehydration	P	P	P
HNZZ-2202	2022	China/Henan	Cat	6 months	Y	High fever, diarrhea	P	P	P
HNZZ-2203	2022	China/Henan	Cat	3 months	Y	High fever, vomit , diarrhea	P	P	P
HNZZ-2204	2022	China/Henan	Cat	5 months	Y	High fever, diarrhea	P	P	P
HNZZ-2301	2023	China/Henan	Cat	4 months	Y	High fever	N	P	P
HNZZ-2302	2023	China/Henan	Cat	3 months	N	High fever, vomit, diarrhea	P	P	P
HNZZ-2303	2023	China/Henan	Cat	3 months	N	High fever, diarrhea	P	P	P

HNZZ-2304	2023	China/Henan	Cat	3 months	Y	High fever, vomit, diarrhea, dehydration	P	P	P
HNZZ-2305	2023	China/Henan	Cat	4 months	Y	High fever, vomit, diarrhea	P	P	P
HNZZ-2306	2023	China/Henan	Cat	3 months	N	High fever	N	P	P
HNZZ-2307	2023	China/Henan	Cat	3 months	N	High fever	N	P	P
HNZZ-2308	2023	China/Henan	Cat	6 months	Y	High fever, vomit, diarrhea, dehydration	P	P	P
HNZZ-2401	2024	China/Henan	Cat	4 months	Y	High fever, diarrhea	P	P	P
HNZZ-2402	2024	China/Henan	Cat	1 months	N	High fever, diarrhea, dehydration	P	P	P
HNZZ-2403	2024	China/Henan	Cat	2 months	N	High fever, vomit, diarrhea, dehydration	P	P	P
HNZZ-2404	2024	China/Henan	Cat	2 months	N	High fever , diarrhea, dehydration	P	P	P
HNZZ-2405	2024	China/Henan	Cat	11 months	Y	High fever	N	P	P
HNZZ-2406	2024	China/Henan	Cat	4 months	Y	High fever, vomit, diarrhea	P	P	P
