# Supplementary material for: Epidemiological and Molecular Investigation of Feline Panleukopenia Virus Infection in China
Source: Viruses. 2024 Dec 23;16(12):1967. doi: 10.3390/v16121967 (PMC11728606; doi:10.3390/v16121967)
Supplement: Supplementary file 1 [file viruses-16-01967-s001.zip › Table S2 ▓╬┐╝╓Ω╡─▒│╛░╨┼╧ó.pdf]

Table S2. Basic information of 39 reference FPV, CPV and MEV strains.

Accession no.	Virus or genotype	Strains	Host	Origin	Year
EU498681.1	FPV	Felocell(Vaccine)	cat	Italy	2008
EF988660.1	FPV	XJ-1	cat	China/Xinjiang	2007
MK295775.1	FPV	JL-3	cat	China/Jilin	2017
MT270536.1	FPV	BJ644	cat	China/Beijing	2019
MT614366.1	FPV	HF1	cat	China/Anhui	2019
OL547736.1	FPV	F-D33	cat	China/Sichuan	2019
MZ836373.1	FPV	JN-FPV-96	cat	China/Shandong	2020
MW811187.1	FPV	SH2003	cat	China/Shanghai	2020
AB054227.1	FPV	V211	cat	Japan	2001
HQ184190.1	FPV	K3	cat	South Korea	2008
OK128324.1	FPV	C-DY6	dog	China/Sichuan	2019
MK357738.1	FPV	HN39AA	dog	Viet Nam	2017
MW035309.1	FPV	19SP_CK-8	dog	South Korea	2019
FJ405225.1	FPV		tiger	China/Jilin	2008
KX685354.1	FPV	HN-ZZ1	tiger	China/Henan	2016
MZ712026.1	FPV	AMPV2020	panda	China/Sichuan	2020
MT178243.1	FPV	SY-01/2019	cheetah	China/Liaoning	2019
MZ005633.1	FPV	HNZZ2	lion	China/Henan	2020
MW926316.1	FPV	Cat 3	cat	United Kingdom	2019
EU659115.1	FPV	kai.us.06	cat	USA	2006
MZ742176.1	FPV	AUS-14	cat	Australia	2017
OM937916.1	FPV	39-566	cat	Egypt	2019
KT240132.1	FPV	PT183/12	cat	Portugal	2012
KX434461.1	FPV	IZSSI_3201_1_15	cat	Italy	2015
FJ712217.1	MEV	Suning	mink	China/Hebei	2008
FJ712221.1	MEV	LYT-2	mink	China/Shandong	2008
GU392252.1	MEV	Dalian2	mink	China/Liaoning	2009
HQ883275.1	MEV	Jlin/2010	mink	China/Jilin	2010
M38245.1	CPV-2	CPV-b	dog	USA	1990
M24003.1	CPV-2a	CPV-15	dog	USA	1998
KC262178.1	CPV-2a	MPCPV-SX	dog	China/Jilin	2012
M74849.1	CPV-2b	39	dog	USA	1995
DQ025992.1	CPV-2b	04S23	dog	France	2005
MF001439.1	New CPV-2a	YZ5	dog	China/Jiangsu	2016
MW017604.1	New CPV-2a	JSYZ-93	dog	China/Jiangsu	2019
MW048564.1	New CPV-2b	JSNT-48	dog	China/Jiangsu	2019
KR611499.1	New CPV-2b	LN-14-2	dog	China/Liaoning	2014
MH711902.1	CPV-2c	CU21	dog	Thailand	2016
MW017624.1	CPV-2c	JSYZ-151	dog	China/Jiangsu	2020
